# Socio-Cultural and Economic Drivers of Plant and Animal Protein Consumption in Malaysia: The SCRiPT Study

**DOI:** 10.3390/nu12051530

**Published:** 2020-05-25

**Authors:** Adam Drewnowski, Elise Mognard, Shilpi Gupta, Mohd Noor Ismail, Norimah A. Karim, Laurence Tibère, Cyrille Laporte, Yasmine Alem, Helda Khusun, Judhiastuty Februhartanty, Roselynne Anggraini, Jean-Pierre Poulain

**Affiliations:** 1 Center for Public Health Nutrition and Department of Epidemiology, University of Washington, Seattle, WA 98195, USA; shilpi24@uw.edu; 2 Faculty of Social Sciences & Leisure Management, Taylor’s University, Subang Jaya 47500, Selangor, Malaysia; eliseline.mognard@taylors.edu.my (E.M.); Ismail.Noor@taylors.edu.my (M.N.I.); yasmine.alem@sd.taylors.edu.my (Y.A.); jean-pierre.poulain@taylors.edu.my (J.-P.P.); 3 International Associated Laboratory-National Center for Scientific Research (LIA-CNRS) “Food Cultures and Health”, 31058 Toulouse, France; tibere@univ-tlse2.fr (L.T.); cyrille.laporte@univ-tlse2.fr (C.L.); 4 International Associated Laboratory-National Center for Scientific Research (LIA-CNRS) “Food Cultures and Health”, Subang Jaya 47500, Malaysia; 5 Centre for Community Health Studies, Faculty of Health Sciences, Universiti Kebangsaan, Kuala Lumpur 50300, Malaysia; norimahkarim@ukm.edu.my; 6 Centre d’Etude et de Recherche: Travail, Organisations, Pouvoir (CERTOP) UMR-CNRS 5044, Axe: “Santé Alimentation”, University of Toulouse 2 Jean Jaurès, 31058 Toulouse, France; 7 SEAMEO Regional Centre for Food and Nutrition (RECFON), Universitas Indonesia, Daerah Khusus Ibukota Jakarta 10430, Indonesia; hkhusun@gmail.com (H.K.); jfebruhartanty@seameo-recfon.org (J.F.); lynne.prigel@gmail.com (R.A.)

**Keywords:** protein transition, animal protein, plant protein, ethnicity, religion, education, incomes, Malaysia, food choices, eating patterns

## Abstract

Countries in South East Asia are undergoing a nutrition transition, which typically involves a dietary shift from plant to animal proteins. To explore the main drivers of protein consumption, the SCRiPT (Socio Cultural Research in Protein Transition) study recruited a population sample in Malaysia (*N* = 1604). Participants completed in-person 24 h dietary recalls and socio-demographic surveys. Energy and nutrient intakes were estimated using Nutritionist Pro. A novel recipe-based frequency count coded protein sources as meat (chicken, beef, pork, and mutton), fish, eggs, dairy, and plants (cereals, pulses, tubers). Dietary intakes and frequencies were examined by gender, age, income, education, ethnicity, religion, and family status, using ANOVAs and general linear models. Energy intakes were 1869 kcal/d for men and 1699 kcal/d for women. Protein intakes were 78.5 g/d for men and 72.5 g/d for women. Higher energy and protein intakes were associated with Chinese ethnicity, higher education and incomes. Frequency counts identified plant proteins in 50% of foods, followed by meat (19%), fish (12%), eggs (12%), and dairy (7%). Most frequent source of meat was chicken (16%) rather than pork or beef (1.5% each). In bivariate analyses, animal protein counts were associated with younger age, higher education and incomes. In mutually adjusted multivariate regression models, animal proteins were associated with education and ethnicity; plant proteins were associated with ethnicity and religion. Protein choices in Malaysia involve socio-cultural as well as economic variables.

## 1. Introduction

Countries in South East Asia are undergoing both economic and nutrition transitions [1]. As incomes rise, diets composed of traditional grain crops are replaced by more animal products, eggs and dairy, and by more processed and fast foods [1,2,3]. Energy from complex carbohydrates falls, to be replaced by dietary energy from added sugars, vegetable oils and animal fats [4,5]. At the same time, dietary plant proteins give way to proteins of animal origin [6].

The shift from plant to animal proteins has been identified as a “protein transition” [6]. Whereas the nutrition transition is viewed as income-driven, the choice of animal protein (meat, fish, or dairy) may also depend on ethnicity, religion, and other social and cultural variables [6]. For that reason, ethnically diverse and increasingly urban Malaysia, now classified among upper-middle income economies by the World Bank, represents a population of particular interest.

Before studies on Malaysia protein transition can be undertaken, a detailed assessment of protein intakes from diverse food sources is required [7,8]. However, there are a number of methodological challenges. Not every nutrient composition database separates dietary protein by food source. In the US-based Seattle Obesity Study, Aggarwal et al. [9] were able to distinguish between animal and plant proteins. Pasiakos et al. [10] used a customized USDA database to separate protein intakes of participants in the National Health and Nutrition Examination Survey (NHANES) into meat, dairy, and plant proteins. Chicken and beef were the primary sources of meat protein; cheese and milk were the primary sources of dairy [10]. By contrast, nutrient composition data for mixed foods by protein source are relatively scarce. Few studies of SE Asia diets have separated different types of plant proteins or distinguished among egg or dairy proteins, and different types of meat, poultry, or fish.

We have developed a novel protein frequency count tool based on recipes. Foods and mixed dishes were scored for the reported presence of protein food sources from 10 categories. The categories were plants (cereals, pulses, tubers), eggs, dairy, meat (beef, pork, mutton, poultry), and fish. A percentage score for protein food sources was derived for each study participant. The frequency counts were applied to all foods listed by participants during an in-person 24 h recall.

The frequency count approach builds on local culinary traditions and the common practice of combining small amounts of plant and animal proteins in a single dish or a single meal. Such complex protein combinations, often driven by tradition or social norms, may be a culturally sanctioned way to avoid amino acid imbalance. However, quantitative nutrient composition data by protein source for many such dishes in Malaysia are not readily available.

Since the consumption of animal-source foods in SE Asia is often viewed as aspirational, the present study asked detailed questions about income, education, and urbanization in addition to ethnicity and religion. Sociodemographic assessments were based on a simplified version of the Malaysian Food Barometer (MFB) [11,12]. Dietary 24 h recalls were the standard method of dietary intake assessment [12,13,14].

## 2. Materials and Methods

### 2.1. Participants

The Malaysian SCRiPT project was based on a nationally representative sample of men and women aged > 18 y. The sampling methodology was based on the Malaysian Food Barometer (MFB1) in 2013 [11]. The MFB1 was based, in turn, on the Malaysian Adult Nutrition Survey (MANS) conducted by the Public Health Institute, Ministry of Health Malaysia in 2003 (*N* = 6928) [15] and 2014 (*N* = 4000) [16]. The SCRiPT stratified random sampling scheme was based on geographic regions within Peninsular Malaysia, Sabah, and Sarawak, taking into account their population size and degree of urbanization. A quota system based on gender, age, and ethnicity was also applied. A subject sampling table was added to help enumerators with respondent selection within the household. The final analytical sample was geographically distributed across the states in Malaysia: Klang Valley or Greater Kuala Lumpur (*N* = 387); Johor (*N* = 188); Sabah (*N* = 163); Sarawak (*N* = 154); Perak (*N* = 138); Kedah (*N* = 122); Penang (*N* = 81); Kelantan (*N* = 90); Pahang (*N* = 96); Terengganu (*N* = 81); Negeri Sembilan (*N* = 54); and Malacca (*N* = 50). Of these, Kuala Lumpur, Johor, Perak, Penang, Negeri Sembilan, and Malacca are more than 50% urban. Sabah, Sarawak, Kedah, Kelantan, and Terengganu are more rural. Sabah and Sarawak are located in East Malaysia. The data were collected using in-person interviews between March and July 2018.

The present sample had more women, younger adults, and urban dwellers than the Malaysian population but was otherwise consistent with the main ethnic distribution in Malaysia as determined by 2010 census. By census estimates, 75% of the Malaysian population is now urban. The ethnic groups are categorized as Malay and non-Malay Bumiputras, Chinese, and Indian. Islam is the largest religion; other groups include Buddhist, Christian, Hindu and Tao.

### 2.2. Data Collection Methods and Procedures

Data were collected using a structured questionnaire in the course of in-person interviews. The questionnaire was pre-tested with a small sample of 37 respondents to ensure that the questions were relevant, well-understood and appropriately asked by the enumerators. The pilot sample was diverse in terms of gender, age, ethnicity, and socioeconomic status. All questions were translated into Malay and Chinese, following protocols established by the Malaysian Food Barometer Project [11]. For Malaysia, the Malay language was reviewed by Drs. Mohd Noor Ismail and Norimah Karim. Chinese language was reviewed by Chinese experts. Questionnaire text and methodology were approved by the Human Ethics Committee of Taylor’s University (reference n° HEC2017/030).

### 2.3. Socio-Demographic Questionnaires

Gender was coded as male, female. Age cutpoints were 18–25 y; 26–35 y, 36–45, and >45 y, roughly corresponding to population quartiles. Income categories were based on the bimodal distribution of continuous data on monthly household incomes in Malaysian Ringgit (RM). Self-reported household income was divided by number of persons in the household to obtain mean monthly income per capita. Low income was defined as <700 RM/month. Middle income was defined as 700–1333 RM/month; high middle income was 1333 to 1999 RM/month; and upper income was >2000 RM/month Education was captured as primary or lower school, lower secondary school, higher secondary school, and college. Options for ethnic origin were Malay, Chinese, Indian, and non-Malay Bumiputra. Options for religious denominations were Muslim, Hindu, Buddhist, Christian, or Taoist. Marital status was single (never married, divorced, separated, widowed) versus married or partnered. Additional questions addressed household size and urbanization.

### 2.4. Dietary Intakes from 24 h Recall

Dietary intake data were collected by face-to-face interview using a structured questionnaire to probe for foods consumed over a 24 h period. SCRiPT participants were asked to list all the food and drinks they consumed in the previous day before the survey from waking up until going to bed again at night. After each food was listed, participants were then asked about the amounts consumed. Reporting of amounts consumed was aided by references to foods listed in the *Album Makanan Malaysia* catalogue [17]. Dietary intake data were then entered into Nutritionist Pro, a Windows software for nutrient intake calculation. The customized software version was based on the 1997 and 2017 versions of the Malaysian Food Composition Table [18]. The Malaysian food database consists of 1892 foods (including prepared foods or recipes), categorized into 14 food groups with 29 mandatory nutrients, including water and energy. The two variables of interest for the SCRiPT study were energy (kcal) and protein (g).

### 2.5. Frequency Count Method for Identifying Protein Source

A novel method to identify protein sources using a frequency count was applied to 24 h recalls. Calculations of the frequency count were based on the following steps. First, in the Nutritionist Pro Software, each food was assigned into one or more protein categories depending on its recipe and on the types of protein. Protein sources were coded first as plant proteins or animal protein. The 3 categories of plant protein were cereals, pulses, and tubers. The 7 categories of animal protein were eggs, dairy, fish, and four kinds of meat: poultry, pork, beef, and mutton.

The recipe-based assignment of protein ingredients into categories depended on dish composition. Standard recipes from the Malaysian Food Composition Table were employed. For example, nasi goreng (fried rice), a frequently eaten composite dish, was assigned to multiple protein categories, including cereals (rice), egg (fried egg), and poultry (chicken) or fish (shrimp or fish cake) or pulses (tofu soy). Depending on the dish composition, the number of protein categories could vary from 0 (no protein) to 7 (every protein category present).

To generate the frequency count metric, the number of counts in each protein source category was summed for each participant. These counts were aggregated into 4 main groups: (1) meat, (2) eggs and dairy, (3) fish, (4) plants and were expressed as percent of total for each participant. Frequency counts were also used to compute the percent of plant-source protein foods and the percent of animal-source protein foods for each participant. It is important to note that the frequency counts were based on 24 h dietary recalls and reflected consumption data. Percentage frequency counts accounted for the presence or absence of animal or plant protein in the food, but not the amount.

### 2.6. Plan of Analysis

**Figure 1:**
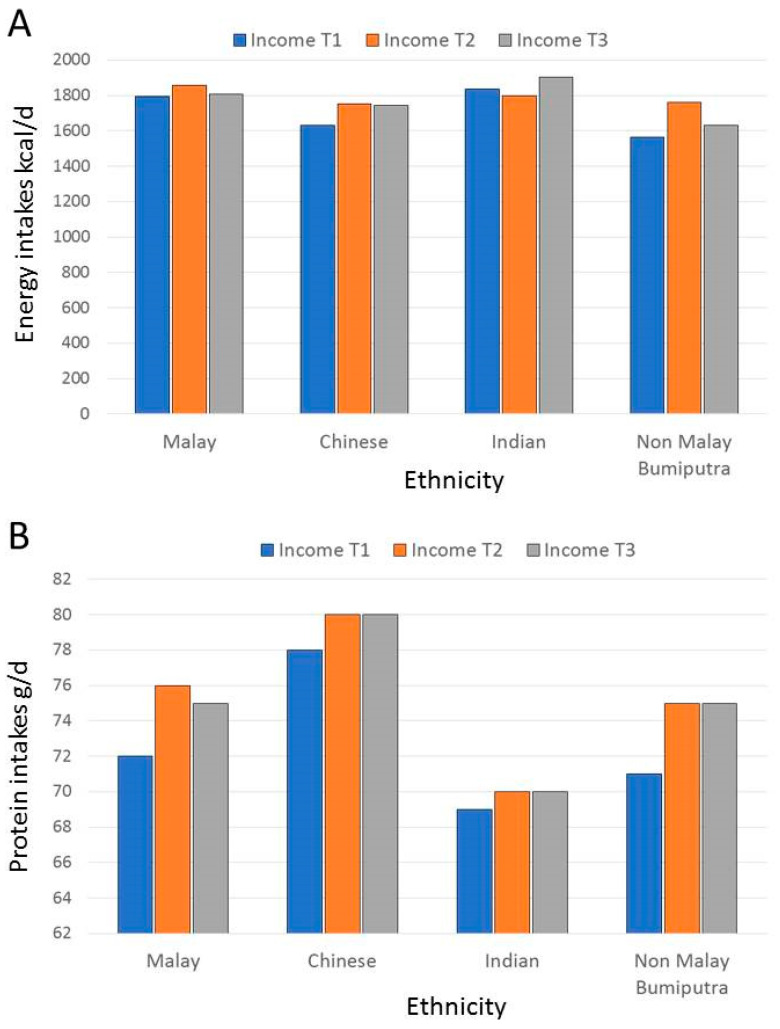
(**A**) Energy intakes (kcal/d) from 24 h recalls by ethnicity and income tertiles (T1, T2, T3). (**B**) Protein intakes (g/day) by ethnicity and income tertiles (T1, T2, T3). Data for Malaysia *n* = 1604.

**Figure 2:**
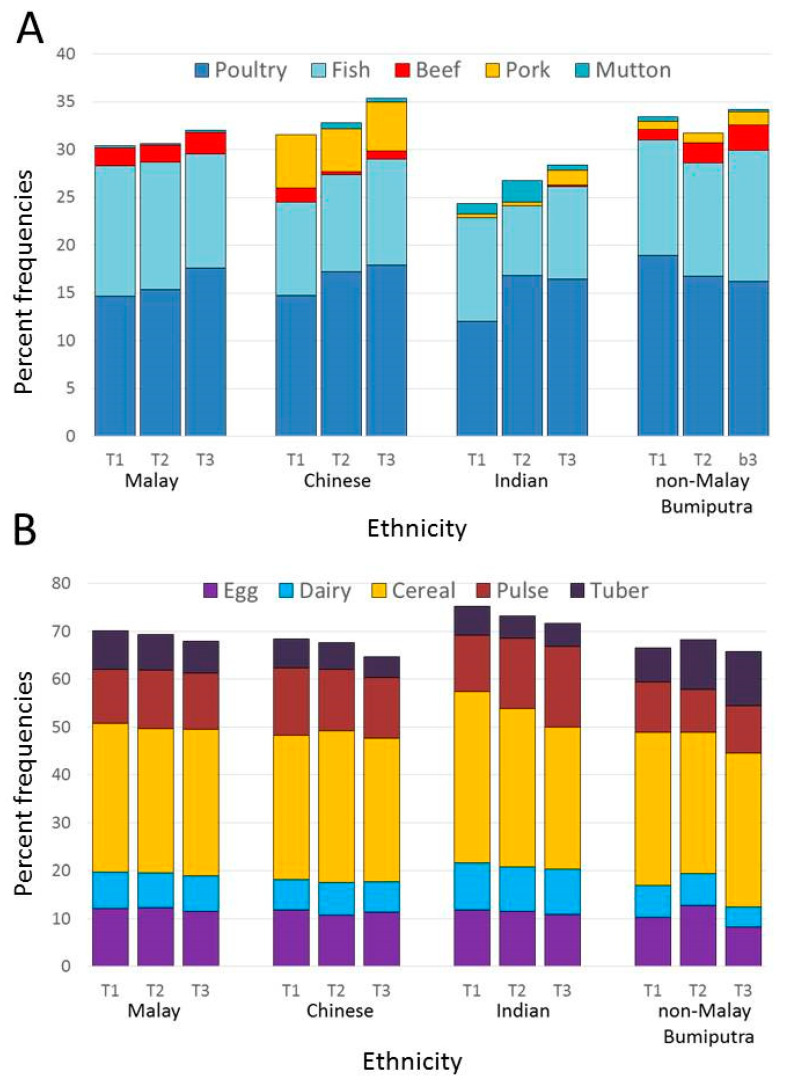
Percentages of different protein sources in 24 h recalls by ethnicity and income tertiles (T1, T2, T3). (**A**) Percent frequencies for poultry, fish, beef, pork and mutton. (**B**) Percent frequencies for eggs, dairy, cereals, pulses and tubers. Data for Malaysia *n* = 1604.

Bivariate tests of differences in protein source frequency counts across sociodemographic groups were based on one-way ANOVA. For cross-analyses of ethnicity by incomes shown in Figure 1 and Figure 2, incomes were recoded into tertiles. Tests of main effects and interactions were based on two-way ANOVA, with tests for significant differences between the means adjusted using the Bonferroni correction. Separate multivariable regression analyses were then conducted for plant and for animal proteins. Strength of the association between socioeconomic variables (education, income, and urbanization) and plant or animal protein counts were tested in multivariable linear regressions with robust standard error, adjusting for cultural variables (ethnicity, religion), and other covariates. Adjustments were conducted using categorical as opposed to continuous variables. Strength of the association between cultural variables and plant or animal protein counts were tested in multivariable linear regressions with robust standard error, adjusting for socio-economic variables and other covariates. Analyses were conducted using SPSS and SAS statistical programs.

## 3. Results

### 3.1. Energy and Protein Intakes from 24 h Dietary Recalls, Malaysia

Table 1 shows the sociodemographic distribution of the sample. Mean energy intake was estimated at 1776 kcal/d (men 1869 kcal/d; women 1699 kcal/d). Mean protein intake was 78.5 g/d for men and 72.5 g/d for women, equivalent to about 17% of dietary energy. Gender effect was significant (*p* < 0.001). Energy intakes peaked for the 25–35 y age group and then declined. No significant age effect was observed for protein intakes. Higher intakes of energy and protein were associated with higher education (*p* < 0.001) and higher intakes of protein were associated with higher incomes (*p* < 0.001). There were also significant effects of ethnicity and religion. Indian respondents had the higher energy intakes but the lowest protein intakes. The Chinese respondents had the highest protein intakes followed by Malay respondents. Buddhist and Taoist religions were also associated with the highest protein intakes in 24 h recall. Marital status had no effect.

Figure 1A shows energy (kcal/d) intakes from 24 dietary recalls by ethnicity and tertiles of monthly income. Income tertile cutpoints were 833 RM (corresponding to 191 US dollars per month) and 1333 RM (corresponsing to 306 US dollars/per month. These analyses were conducted using two-way ANOVA. For energy intakes (kcal/d), there was a significant main effect of ethnicity (F(31,592) = 6.51; *p* < 001)but no main effect of income and no interaction.

Figure 1B shows protein intakes (g/d) from 24 dietary recalls by ethnicity and tertiles of income. There was a significant main effect of ethnicity (F(21,592) = 3.861; *p* < 0.01) but no effect of income and no interaction. The highest protein intakes were observed among Chinese respondents; their protein intakes were significantly higher than those of Malay respondents (*p* < 0.01), Indian respondents (*p* < 0.05) and non Malay Bumiputra respondents (*p* < 0.5). The effect of icome was not significant and there was no significant interaction.

### 3.2. Plant and Animal Protein Frequency Counts from Recipes in 24 h Recalls

Table 2 shows percent protein source frequency counts obtained from recipes for foods listed in 24 h recalls. First, there was a 50:50 split between plant and animal proteins. Plant proteins were listed in 49.69% of foods consumed, whereas animal proteins were listed in 50.31% of foods consumed. Animal proteins were then disaggregated into meat proteins (19.43%), eggs and dairy (18.76%), and fish (12.13%).

There was a significant effect of education. Higher counts for plant proteins were associated with lower education. Higher counts for animal proteins were associated with higher education (*p* < 0.001 for both). Higher frequency counts for fish (*p* = 0.03) and meat (*p* < 0.001) were also associated with higher education. Higher frequency of meat consumption was also associated with higher incomes.

There were significant effects of ethnicity. The highest frequencies for plant proteins were associated with Indian and non-Malay Bumiputra respondents. The lowest frequencies for animal proteins were also associated with Indian and non-Malay Bumiputra respondents (*p* < 0.001 for both). The highest frequencies of eggs and dairy were reported by Indian respondents, the highest frequencies of fish by Malay respondents and the highest frequencies of meat by Chinese respondents.

There were strong effects of religion. The highest frequencies of plant proteins were reported by Hindu respondents. The lowest frequencies of animal proteins were also reported by Hindu respondents. The highest frequencies for meat were reported by Christian and Buddhist respondents and the highest frequencies for fish by Malay respondents. Reported plant and animal protein frequencies were not affected by marital status or urbanization.

Table 3 shows percent frequency counts for 24 h recalls for fish, poultry, beef, pork, and mutton. In general, poultry (16.25%) was listed more frequently than fish (12.12%). Beef (1.47%), pork (1.41%), and mutton (0.30%) were components of very few foods or dishes. Poultry was associated with younger age groups (*p* < 0.008) and with higher education (*p* < 0.001). By contrast, fish and pork were associated older age groups (*p* < 0.003). There were significant effects of ethnicity: Fish and beef were associated with Malay respondents (*p* < 0.001), whereas pork was associated with Chinese respondents (*p* < 0.001). Frequencies of pork and poultry (but not fish) consumption were a function of incomes (*p* < 0.001).

Percent protein counts for.fish were about 12% of the total. Mean reported frequencies for beef and pork were about 1.5% and for mutton less than 1%. Mean reported frequencies for poultry (chicken) were approximately 16% of the total. There were significant differences in frequency counts by ethnicity and income tertiles. For meat and fish frequencies, there was a significant main effect of ethnicity (F(31,592) = 10.694; *p* < 0.001), a main effect of income (F(31,592) = 4.043; *p* < 0.05) but no interaction. For fish, there was a main effect of ethnicity (F(31,592) = 9.829; *p* < 0.001) but no effect of income and no interaction. Significant effects of ethnicity were also observed for beef, pork and mutton (*p* < 0.001 for all). By contrast, no effect of ethnicity and no effect of income were observed for poultry, which is less subject to social and religious influences.

Malay Bumiputra respondents had higher protein counts for fish as compared to Indian (*p* < 0.001) and Chinese respondents (*p* < 0.001). Non-Malay Bumiputra respondents had higher fish protein counts as compared to Indian respondents (*p* < 0.05). Bumiputra Malay respondents had higher protein counts for beef as compared to Indian (*p* < 0.001) and Chinese respondents (*p* < 0.001). Non-Malay Bumiputra respondents had higher beef protein counts as compared to Indian respondents (*p* < 0.05). and Chinese respondents (*p* < 0.05). Chinese respondents high higher frequency counts for pork as compared to Malay, non-Malay Bumiputra and Indian respondents (*p* < 0.05 for all). For mutton, there was a significant main effect of ethnicity (F(31,592) = 10.603; *p* < 0.001) and an ethnicity by income interaction (F(61,592) = 3.240; *p* < 0.005). Indian respondents listed muton more frequently than did Malay, non Malay Bumiputra and Chinese respondents.

Figure 2B shows percent protein frequency counts for eggs, dairy, cereals, pulses, and tubers. For the totals presented, there were significant main effects of ethnicity (F(31,592) = 10.620; *p* < 0.001) and income (F(21,592) = 3.994; *p* < 0.05) but no interaction. For egg and dairy, there was a significant effect of ethnicity (F(31,592) = 6.251; *p* < 0.001), but a weak effect of income(*p* = 0.061) and no interaction. For dairy, there was a significant effect of ethnicity (F(31,592) = 5.777; *p* < 0.001), no effect of income and no interaction. Indian respondents were more likely to list dairy than were Chinese or non-Malay Bumiputra respondents. For plant proteins from cereals, tubers, and pulses, there was no effect of income and no interaction.

### 3.3. Multivariate Regression Analyses

Table 4 shows the results of multivariate regression with the frequency of animal protein intake and plant protein intake as the dependent variables. Model 1 adjusted for basic sociodemographics: age, gender, and marital status. Model 2 mutually adjusted SES variables (education and incomes) for ethnicity and religion and and the social variables for SES. The goal was to establish which factors predict the consumption of animal proteins versus plant proteins. Percent frequency counts were the dependent variables.

For animal protein frequency, there were strong effects in Model 1 of ethnicity and education, but not religion and a weak effect of income. The highest frequency of animal protein was reported by educated Malay participants. The same results were obtained in Model 2. For plant protein frequency, there were strong effects of ethnicity and religion but not of education or income.

## 4. Discussion

The present results were consistent with past research on the quality and characteristics of the Malaysian diet. The present estimates of energy intakes from 24 h dietary recalls were 1869 kcal/d for men and 1699 kcal/d for women, consistent with earlier reports. A previous Malaysia Nutrition Survey MANS 2008 [19] placed energy intakes at 1776 kcal/d for men and 1447 kcal/d for women. Kaur et al., 2016 [20] estimated median energy intakes in 101 Malaysian Punjabi adults at 1826 kcal/d, also using 24 h recall. Lee & Muda, 2019 [21] reported energy intake of adults residing in two cities in Malaysia (Penang, Kota Bharu) at 1664 kcal/d for men and 1491 kcal/d for women.

The present protein values from 24 h recalls were 78.5 g/d for men and 72.5 g/d for women, in excess of recommendations (50 g/d). Previously obtained median protein intakes were 69.8 g/d [21]. Lee and Muda (2019) [21] obtained mean values of 73.4 g/d for men and 67.1 g/d for women. Another Malaysia-based study [22] reported energy intakes of 128 women living in Kuala Lumpur at around 1800 kcal/d and protein intakes at 71 g/d.

The work on improving nutrient composition datasets in Malaysia is ongoing. However, there are few practical options for assessing protein intakes by food source from 24 h dietary recalls using standard nutrient composition tables. This study combined 24 h dietary recalls with frequency counts of diverse protein food sources. To obtain frequency counts, foods and recipes were scored for the presence of different types of animal and/or plant proteins. While the presence of different proteins was taken into account, the relative amounts consumed were not. Nonetheless, a simple count of what types of dietary proteins were a part of 24 h dietary recalls served as a novel protein diversity score. There are obvious parallels to the Food and Agriculture Organization dietary diversity scores [23], which also assessed habitual diets for the presence/absence of a food group but did not assess the amounts consumed. In prior research, dietary diversity scores have been validated as proxy measures for macro and/or micronutrient adequacy of the diet for several vulnerable populations of interest [23].

Malaysian eating patterns differ from one state to another; the main differences occur between Peninsular Malaysia and East Malaysia due to multiple socio-economic and socio-cultural factors. Whereas Peninsular Malaysia is becoming urban, East Malaysia states Sabah and Sarawak are rural. The present analyses did not find a strong overall effect of urbanization on the percent frequencies of total animal proteins. However, the effects of urbanization may differ by incomes and ethnicity and also by the type of animal protein. Urban households may consume more meat, especially chicken whereas the diets of rural household may feature the more traditional fish. The recipe-based method of frequency counts, while relatively crude, identified Malay participants as most likely to consume beef and Chinese participants as most likely to consume pork. Indian participants were less likely to consume either one, and had the lowest total protein intakes.

The present data point to the complexity of protein choices once the different food sources of protein are identified. First, the proportion of plant versus animal protein food sources was around 50:50. We would expect the proportion of plant protein food sources to be much higher in low- and middle-income countries in South East Asia, such as Indonesia. [24] Second, it was interesting to note the presence of chicken rather than fish among participants with higher education and incomes. Those participants were also more likely to include eggs and dairy in dishes. The reported frequencies for proteins from beef and pork in dishes were extremely low, especially in comparison to chicken and fish. When it came to plant proteins, cereals (mostly rice) were reported more frequently than were pulses or tubers.

The present approach, based on recipe-based frequency counts of protein sources, may find use in those studies where nutrient composition data by protein source are not available. In general, dietary variety has served as a proxy index of good nutrition. Based on dietary diversity findings, the FAO recommends achieving amino acid balance by supplementing grain and cereal based diets in low income countries with small amounts of high-quality protein. Mixing multiple protein sources in a single dish results in improved nutrition but can pose problems for dietary intake assessment in nutritional epidemiology. Our frequency score is an innovation as it captures dietary variety in animal and plant protein sources.

The present combination of 24 h recalls supplemented with protein source frequency counts and analyzed using multiple regression models was relatively rigorous. A recent study on the transition to animal proteins in India [25] assigned respondents to different type diets (lacto vegetarian, ovolacto vegetarian with egg, ovolacto vegetarian without egg, chicken diet and meat diet) on the basis of their responses to 29 questions. Analyses then compared percentages of each diet by caste, region, gender, and age group. No statistics were provided [25]. Although the present approach also relies on reported consumption frequencies, those data need to be supplemented with some quantitative analyses.

The data suggest that the current patterns of protein consumption in Malaysia, as ascertained by a combination of 24 h dietary recalls and the recipe-based protein frequency count, were influenced by more than incomes. The present analyses identified education, ethnicity and religion, rather than monthly incomes, among the drivers of protein choice. The impact of ethnicity was significant when it came to the inclusion in the diet of fish, beef, pork or mutton. By contrast, the inclusion of chicken was less influenced by sociocultural variables.

The present analyses showing that ethnicity and religion influence the choice of plant versus animal proteins in Malaysia need to be discussed in the context of the SE Asia nutrition transition [6]. Thus far, the nutrition transition occurring in low- and middle-income countries has been viewed as an inevitable consequence of economic development [2]. As incomes rise, traditional food patterns built around starchy staples (cereals, roots, and tubers) gradually give way to more animal-source foods [1]. Plant-source proteins decline and are replaced by proteins of animal origin [1,2]. In SE Asia, the consumption of rice has declined, whereas dietary energy from added sugars and fats has increased. The present data are consistent with other reports on “compressed modernization” [12,13,26] showing that that plant proteins in Malaysia were being replaced by proteins from red meat, chicken, eggs and dairy. In the present study, the choice of animal protein (beef, pork, mutton or fish) was driven not so much by incomes but by ethnicity and culture.

Paradoxically, the ongoing trend toward more animal proteins in Malaysia and other SE Asian countries has now been countered by the trend toward more plant proteins in high income countries. Effectively, two opposing protein transitions are under way. As low and middle income countries are increasing the consumption of animal source foods, high income countries are promoting the opposite. The plant-forward diets of rich countries will contain no red meat, or processed meat and only low to moderate amounts of seafood and poultry [27]. The healthy reference diet for the planet, as recommended by the EAT Lancet Report [27], will consist of vegetables, fruits, whole grains, legumes, and nuts, with little or no added sugar, refined grains, potatoes, or corn. It remains to be determined whether this opposing protein transition is driven by incomes or does it depend on local convictions, ideologies, and culture.

The study had limitations. Analyses of the complex relations between food and eating patterns by geographic location are not included in this paper. Second, the present estimates of food consumption were based on a single 24 h dietary recall. That method can provide an estimate of energy and nutrient intakes for groups but not for individuals. Third, the protein source frequency count provided an index of protein diversity without providing the amount. As such, the present method had more in common with a diversity index than with a food frequency questionnaire. Still the method appeared to be sensitive to variations by economic and social strata. In the absence of detailed nutrient composition databases by protein food source, frequency counts may be a useful addition to the methods toolbox.

## 5. Conclusions

Protein choices in Malaysia involve socio-cultural as well as economic variables. Percent frequencies for animal versus plant proteins were influenced by education, ethnicity and religion, rather than incomes only. There were also specific differences by animal protein type. Among younger and better educated adults, chicken was listed more frequently than fish, the traditional protein staple [26]. Such data can provide some insight as to the nature of dietary choices in multi-cultural and multi-religious societies such as Malaysia. Animal products are the main food category that is subject to religious taboos or cultural norms. Follow up studies will explore the social aspects of protein transition in Malaysia.

## Figures and Tables

**Table 1:** Energy and protein intakes from 24 h recalls (*N* = 1604) by socio-demographics. Data are means and standard errors of the mean (SEM). *p*-values are for main effects using one-way ANOVA.

Socio-Demographic Strata	Malaysia		Energy (kcal/d)		Protein (g/d)	
	Count	Percent	Mean	SEM	Mean	SEM
All	1604	100	1776.4	13.9	75.22	0.68
Gender						
Male	729	45.4	1869.2	20.6	78.48	0.98
Female	875	54.6	1699.1	18.5	72.50	0.93
*p-value* ^1^			0.000 ***		0.000 ***	
Age groups (y)						
18–25	441	27.5	1807.2	27.4	75.41	1.29
26–35	440	27.4	1815.8	27.0	76.82	1.34
36–45	312	19.5	1729.7	29.18	72.92	1.42
46+	411	25.6	1736.7	27.5	74.72	1.36
*p-value* ^1^			0.050 *		*0.255*	
Ethnicity						
Malay	888	55.4	1820.2	18.8	74.30	0.90
Chinese	390	24.3	1727.5	28.1	79.07	1.35
Indian	119	7.4	1850.6	48.8	71.33	2.12
Non-Malay Bumiputra	207	12.9	1638.0	37.5	72.86	2.10
*p-value* ^1^			0.000 ***		0.001 ***	
Monthly income per person (Malaysian Ringgit)						
RM < 700	357	22.3	1703.9	30.0	68.59	1.42
RM 700–1333	700	43.6	1810.4	20.1	77.18	0.99
RM 1333–2000	237	14.8	1757.3	35.8	76.18	1.79
RM > 2000	310	19.3	1797.7	33.9	77.69	1.59
*p-value* ^1^			0.025 **		0.000 ***	
Highest level of education						
Primary or lower	124	7.7	1679.5	50.8	71.16	2.22
Lower secondary school	260	16.2	1721.5	33.0	72.44	1.66
Upper secondary school	714	44.5	1765.4	20.9	73.83	1.01
College/ University	506	31.5	1843.8	24.8	79.59	1.22
*p-value* ^1^			0.003 ***		0.000 ***	
Religion						
Muslim	996	62.1	1795.6	17.6	74.06	0.86
Buddhist	256	16.0	1733.8	34.6	79.72	1.69
Christian	206	12.8	1688.6	39.4	74.32	1.97
Hindu	99	6.2	1857.8	54.8	71.82	2.42
Taoism	47	2.9	1814.1	80.1	86.29	3.78
*p-value* ^1^			0.038 **		0.001 ***	
Marital status						
Single	815	50.8	1799.5	19.9	74.99	0.92
Married or partnered	789	49.2	1752.6	19.5	75.45	0.98
*p-value* ^1^			*0.92*		*0.735*	

^1^ One-Way ANOVA; * 0.05 < *p* < 0.1; ** 0.01 < *p* < 0.05; *** 0.000 < *p* < 0.01.

**Table 2:** Reported percent frequencies of protein food sources from 24 h intakes by socio-demographics. Protein food sources are shown as plant, total animal, eggs and dairy, fish, and meat. Data are means and standard errors (SEM. *p*-values are for univariate analyses based on one-way ANOVA.

Socio- Demographic Strata		Plant Proteins		Animal Proteins							
				Total Animal		Egg and Dairy		Fish		Meat	
	N	Mean (%)	SEM	Mean (%)	SEM	Mean (%)	SEM	Mean (%)	SEM	Mean (%)	SEM
All	**1604**	**49.69**	**0.31**	**50.31**	**0.31**	**18.76**	**0.27**	**12.13**	**0.23**	**19.43**	**0.27**
Gender											
Male	729	49.64	0.44	50.35	0.44	19.37	0.40	11.33	0.32	19.64	0.40
Female	875	49.72	0.44	50.27	0.44	18.24	0.37	12.79	0.33	19.24	0.37
*p-value* ^1^		*0.896*		*0.896*		0.041 **		0.002 ***		*0.467*	
Age Groups											
18–25	441	48.03	0.55	51.96	0.55	20.63	0.54	10.87	0.44	20.45	0.51
26–35	440	49.46	0.58	50.53	0.58	18.74	0.49	12.32	0.44	19.47	0.51
36–45	312	49.54	0.72	50.45	0.72	19.17	0.62	12.12	0.52	19.16	0.60
46 and above	411	51.81	0.65	48.18	0.65	16.44	0.53	13.27	0.47	18.47	0.57
*p-value* ^1^		0.000 ***		0.000 ***		0.000 ***		0.003 ***		0.070 *	
Ethnicity											
Malay	888	49.74	0.40	50.24	0.40	19.39	0.36	13.10	0.30	17.76	0.34
Chinese	390	48.45	0.65	51.54	0.65	17.73	0.53	10.61	0.48	23.19	0.56
Indian	119	52.45	1.25	47.54	1.25	20.86	1.09	9.42	0.85	17.25	1.01
Non-Malay Bumiputra	207	50.17	0.91	49.82	0.91	16.76	0.80	12.34	0.66	20.70	0.79
*p-value* ^1^		0.020 **		0.019 **		0.001 ***		0.000 ***		0.000 ***	
Monthly income per person (Malaysian Ringgit)											
RM < 700	357	50.76	0.70	49.23	0.70	19.57	0.61	12.48	0.50	17.17	0.56
RM 700–1333	700	49.86	0.54	50.13	0.45	18.78	0.41	12.45	0.34	18.92	0.41
RM 1333–2000	237	49.02	0.73	50.97	0.73	18.47	0.71	11.38	0.64	21.11	0.70
RM > 2000	310	48.57	0.77	51.42	0.77	17.99	0.59	11.56	0.53	21.86	0.63
*p-value* ^1^		*0.119*		*0.119*		*0.304*		*0.270*		0.000 ***	
Highest level of education											
Primary or lower	124	53.47	1.29	46.52	1.29	17.50	1.07	10.95	0.83	18.07	0.96
Lower secondary school	260	49.62	0.81	50.37	0.81	19.80	0.67	11.83	0.53	18.70	0.66
Upper secondary school	714	49.88	0.45	50.11	0.45	18.33	0.41	12.87	0.36	18.91	0.41
College/University	506	48.52	0.53	51.47	0.53	19.12	0.46	11.49	0.40	20.85	0.48
*p-value* ^1^		0.001 ***		0.001 ***		*0.132*		0.030 **		0.004 ***	
Religion											
Muslim	996	49.98	0.37	50.01	0.37	18.96	0.34	13.08	0.29	17.97	0.33
Buddhist	256	48.10	0.78	51.89	0.78	17.73	0.63	11.09	0.62	23.06	0.72
Christian	206	48.18	0.92	51.81	0.92	18.91	0.77	10.63	0.61	22.27	0.77
Hindu	99	52.63	1.42	47.36	1.42	20.23	1.23	9.42	0.88	17.70	1.09
Taoism	47	52.41	2.30	47.58	2.30	16.28	1.64	9.78	1.16	21.52	1.55
*p-value* ^1^		0.004 ***		0.004 ***		*0.149*		0.004 ***		0.000 ***	
Marital status											
Single	815	49.04	0.42	50.95	0.42	19.69	0.39	11.35	0.31	19.90	0.38
Married/Partnered	789	50.36	0.46	49.63	0.46	17.79	0.37	12.93	0.34	18.92	0.39
*p-value* ^1^		0.035 **		0.035 **		0.001 ***		0.001 ***		0.074 *	

^1^ One-Way ANOVA; * 0.05 < *p* < 0.1; ** 0.01 < *p* < 0.05; *** 0.000 < *p* < 0. Consumption patterns of plant and animal proteins showed different socio-demographic profiles. First, there was a significant effect of age. Higher counts for plant proteins were associated with older age groups, whereas higher counts for animal proteins were associated with younger age groups. Higher frequency counts for fish were associated with older adults, whereas higher frequency counts for eggs and dairy were associated with younger adults (*p* < 0.001 for all).

**Table 3:** Reported percent frequencies of animal protein food sources from 24 h recalls by socio demographics. Data are means and standard errors of the mean (SEM). *p*-values are for main effects based on one-way ANOVA.

Socio-Demographic Strata	Malaysia	Fish		Beef		Pork		Mutton		Poultry	
	N	Mean (%)	SEM	Mean (%)	SEM	Mean (%)	SEM	Mean (%)	SEM	Mean (%)	SEM
All	**1604**	**12.12**	**0.23**	**1.47**	**0.10**	**1.41**	**0.11**	**0.30**	**0.50**	**16.25**	**0.26**
Gender											
Male	729	11.33	0.32	1.62	0.15	1.60	0.17	0.29	0.74	16.12	0.38
Female	875	12.79	0.33	1.34	0.13	1.25	0.14	0.31	0.67	16.36	0.36
*p-value* ^1^		0.002 ***		*0.163*		*0.121*		*0.876*		*0.641*	
Age groups (y)											
18–25	441	10.87	0.44	1.69	0.19	0.95	0.15	0.42	0.12	17.38	0.49
26–35	440	12.32	0.44	1.56	0.20	1.24	0.19	0.28	0.08	16.37	0.50
36–45	312	12.12	0.52	1.42	0.22	1.21	0.23	0.26	0.09	16.26	0.58
46+	411	13.27	0.47	1.18	0.18	2.22	0.29	0.23	0.92	14.90	0.54
*p-value* ^1^		0.003 ***		*0.297*		0.000 ***		*0.52*		0.008 ***	
Ethnicity											
Malay	888	13.10	0.31	1.91	0.15	0.00	0.00	0.22	0.05	15.65	0.34
Chinese	390	10.61	0.48	0.78	0.16	5.04	0.38	0.22	0.10	17.14	0.55
Indian	119	9.42	0.85	0.05	0.05	0.81	0.29	1.21	0.37	15.16	0.96
Non-Malay Bumiputra	207	12.34	0.66	1.69	0.28	0.96	0.21	0.28	0.12	17.76	0.75
*p-value* ^1^		0.000 ***		0.000 ***		0.000 ***		0.000 ***		0.011 **	
Average monthly income per person (Malaysian Ringgit)											
RM < 700	357	12.48	0.50	1.77	0.26	0.69	0.15	0.36	0.12	14.34	0.54
RM 700–1333	700	12.45	0.34	1.25	0.13	1.13	0.14	0.26	0.06	16.31	0.39
RM 1333–2000	237	11.38	6.41	1.89	0.28	2.16	0.35	0.37	0.17	16.67	0.67
RM > 2000	310	11.56	0.53	1.28	0.20	2.29	0.33	0.27	0.10	18.01	0.64
*p-value* ^1^		*0.27*		0.064 *		0.000 ***		*0.799*		0.000 ***	
Highest level of education											
Primary or lower	124	10.95	0.83	0.86	0.25	2.02	0.46	0.35	0.24	14.83	0.89
Lower secondary school	260	11.86	0.53	1.33	0.25	1.58	0.31	0.27	0.11	15.51	0.63
Upper secondary school	714	12.87	0.36	1.58	0.16	1.25	0.15	0.29	0.07	15.81	0.39
College/University	506	11.49	0.40	1.53	0.17	1.38	0.19	0.32	0.84	17.60	0.48
*p-value* ^1^		0.03 **		*0.29*		*0.307*		*0.979*		0.005 ***	
Religion											
Muslim	996	13.08	0.29	1.93	0.14	0.06	0.02	0.22	0.04	15.78	0.33
Buddhist	256	11.09	0.62	0.79	0.20	5.15	0.47	0.33	0.16	16.77	0.69
Christian	206	10.63	0.60	1.04	0.24	2.64	0.39	0.37	0.14	18.20	0.74
Hindu	99	9.42	0.88	0.06	0.67	0.98	0.36	1.10	0.40	15.54	1.04
Taoism	47	9.78	1.16	0.17	0.17	5.01	1.07	0.00	0.00	16.32	1.61
*p-value* ^1^		0.000 ***		0.000 ***		0.000 ***		0.001 ***		0.041 **	
Marital status											
Single	815	11.35	0.31	1.50	0.13	1.23	0.14	0.28	0.70	16.87	0.37
Married/Partnered	789	12.93	0.34	1.43	0.14	1.59	0.17	0.32	0.07	15.61	0.37
*p-value* ^1^		0.001 ***		*0.737*		*0.111*		*0.699*		0.017 **	

^1^ One-Way ANOVA; * 0.05 < *p* < 0.1; ** 0.01 < *p* < 0.05; *** 0.000 < *p* < 0.01. Figure 2A shows percent protein frequency counts for poultry, fish, beef, pork, and mutton.by ethnicity and income tertiles (T1, T2, T3). Two-way ANOVA of percent frequency counts for total animal protein showed a weak effect of ethnicity (F(31,592) = 2.429; *p* = 0.064) but no main effect of income and no interaction.

**Table 4:** Multivariable linear regression analyses to test the strength of associations between economic and socio-cultural variables and percent plant and animal protein frequencies.

		Model 1			Model 2		
	N	Coef	*p*-Value	95% CI	Coef	*p*-Value	95% CI
Animal protein							
Monthly income per person (Malaysian Ringgit)							
RM < 700	357	Ref			Ref		
RM 700–1333 RM 1332.99	700	0.38	<0.05	0.06, 0.70	0.36	<0.05	0.04, 0.68
RM 1333–2000	237	0.06	0.757	−0.35, 0.48	0.08	0.722	−0.34, 0.49
RM > 2000	310	0.19	0.342	−0.20, 0.58	0.23	0.270	−0.18, 0.63
Highest level of education							
Primary or lower	124	Ref			Ref		
Lower secondary school	260	0.74	<0.01	0.20, 1.28	0.72	<0.01	0.18, 1.26
Upper secondary school	714	0.59	<0.059	0.10, 1.08	0.57	0.05	0.08, 1.06
College/University	506	0.89	<0.005	0.37, 1.41	0.89	<0.001	0.37, 1.41
Religion							
Muslim	996	Ref			Ref		
Buddhist	256	0.15	0.404	−0.20, 0.49	0.14	0.427	−0.20, 0.48
Hindu	99	0.21	0.420	−0.30, 0.73	0.22	0.404	−0.30, 0.74
Christian	206	−0.29	0.129	−0.66, 0.09	−0.28	0.135	−0.66, 0.09
Taoism	47	−0.57	0.128	−1.30, 0.16	−0.55	0.141	−1.3, 0.18
Ethnicity							
Malay	888	Ref			Ref		
Chinese	390	−0.32	<0.05	−0.62, −0.02	−0.28	0.09	−0.59, 0.04
Indian	119	−1.00	<0.000	−1.48, −0.53	−1.000	<0.000	−1.48, −0.53
Bumiputra	207	−0.63	<0.001	−1.00, −0.25	−0.62	<0.001	−0.99, −0.24
Plant protein							
Monthly income per person (Malaysian Ringgit)							
RM < 700	357	Ref			Ref		
RM 700–1333	700	0.32	<0.05	0.07, 0.57	0.32	<0.05	0.07, 0.57
RM 1,333–2000 RM 1999.99	237	−0.01	0.965	−0.33, 0.32	0.07	0.660	−0.25, 0.40
RM > 2000	310	0.00	0.982	−0.30, 0.31	0.15	0.351	−0.17, 0.47
Highest level of education							
Primary or lower	124	Ref			Ref		
Lower secondary school	260	0.22	0.507	−0.28, 0.57	0.15	0.497	−0.28, 0.57
Upper secondary school	714	0.20	0.636	−0.29, 0.48	0.09	0.633	−0.29, 0.48
College/University	506	0.21	0.337	−0.21, 0.61	0.22	0.300	−0.19, 0.62
Religion							
Muslim	996	Ref			Ref		
Buddhist	256	0.02	0.889	−0.25, −0.29	0.02	0.894	−0.25, 0.29
Hindu	99	−1.00	0.000	−1.41, −0.60	−0.97	<0.000	−1.38, −0.57
Christian	206	0.01	0.932	−0.28, 0.31	0.01	0.939	−0.28, 0.30
Taoism	47	−0.55	0.059	−1.12, −0.02	−0.53	0.07	−1.11, 0.04
Ethnicity							
Malay	888	Ref			Ref		
Chinese	390	−0.49	<0.000	−0.73, −0.25	−0.46	<0.000	−0.71, −0.22
Indian	119	−0.41	<0.05	−0.79, −0.04	−0.40	<0.05	−0.77, −0.02
Bumiputra	207	−0.51	<0.001	−0.81, −0.22	−0.50	<0.001	−0.80, −0.20

Model 1: adjusted for gender, age, and marital status. Model 2: Economic variables (education, income) and socio-cultural varaibles (ethnicity, religion) mutually adjusted for each other.
